# Identification of RNase-sensitive LINE-1 Ribonucleoprotein Interactionsby Differential Affinity Immobilization

**DOI:** 10.21769/BioProtoc.3200

**Published:** 2019-04-05

**Authors:** Hua Jiang, Martin S. Taylor, Kelly R. Molloy, Ilya Altukhov, John LaCava

**Affiliations:** 1 Laboratory of Cellular and Structural Biology, The Rockefeller University, New York, USA; 2 Department of Pathology, Massachusetts General Hospital, Boston, USA; 3 Laboratory of Mass Spectrometry and Gaseous Ion Chemistry, The Rockefeller University, New York, USA; 4 Moscow Institute of Physics and Technology, Dolgoprudny, Russia

**Keywords:** Retrotransposon, LINE-1, L1 RNP, Affinity capture, RNase treatment

## Abstract

Long Interspersed Nuclear Element-1 (LINE-1, L1) constitutes a family of autonomous, self-replicating genetic elements known as retrotransposons. Although most are inactive, copious L1 sequences populate the human genome. L1s proliferate in a ‘copy-and-paste’ fashion through an RNA intermediate; a full-length L1 transcript is ~6,000 nucleotides long and functions as a bicistronic mRNA that encodes and assembles *in cis* with two main polypeptides, ORF1p and ORF2p, forming a ribonucleoprotein (RNP); L1 RNPs also interact with a wide range of host factors in positive and negative regulatory capacities. The following protocol describes an approach to affinity enrich ectopically expressed L1 RNPs and, using RNases, release the fraction of protein that depends upon the presence of intact RNA for retention in the immobilized macromolecules.

## Background

**Figure 1.**
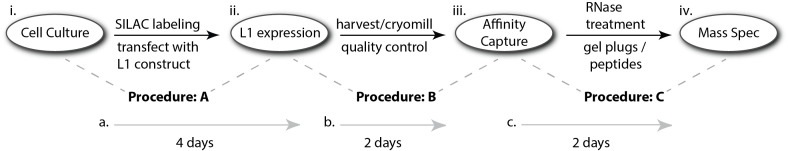
Schematic diagram of this protocol. (i.) Cells will be grown in heavy and light SILAC media and will be transfected with a plasmid supporting (ii.) Inducible, ectopic L1 expression. From the moment the cells reach sufficient density to execute the transfection, (a.) it will be ~4 more days to harvest and freeze them; this is a natural stopping point in the protocol. These manipulations are described in Procedure A. (b.) An additional ~2 days will be needed to cryomill the cells to powder and carry out quality control checks by western blot and other protein assays, as well as to complete the peptide workup for mass spectrometry (MS), to determine the degree of heavy SILAC-label incorporation. The MS analysis could be completed as quickly as ~1 more day if done immediately. These manipulations are described in Procedure B. Pause until thorough SILAC labeling is confirmed. (iii.) Carry out affinity capture, followed by RNase and control treatments; these manipulations are described in Procedure C. (c.) Affinity capture and subsequent peptide workup (described in Procedure D) will require ~2 days. The time to complete (iv.) MS (Procedure D) and data analysis will depend on the number of samples but could be done within an additional ~3 days if the experiment is designed exactly as laid out in this protocol. Aside from the LC-MS/MS runs themselves, a substantial portion of the time is occupied by processing the RAW data files in MaxQuant; speed will vary according to the capabilities of the workstation.

In previous work, an inducible system for the ectopic expression of human L1 in human embryonic kidney (HEK) cells was established. This was achieved by (1) genomically integrating the reverse tetracycline-dependent transactivator (rtTA; Gossen *et al.*, 1995) from the Tet-On Advanced system (Clontech) into the cell line HEK-293T (Dai *et al.*, 2012); the Tet-On modified HEK cell line (referred to originally as HEK-293T-tet-on_LD_, and subsequently as HEK-293T_LD_) was then (2) transfected with episomal vectors (pCEP4 series, Invitrogen; Ostertag *et al.*, 2000; An *et al.*, 2009) containing a tetracycline response element promoter (pTRE-Tight, Clontech), which permitted (3) expression of the L1 cassette in response to the presence of doxycycline in the culture medium (Dai *et al.*, 2012; O'Donnell *et al.*, 2013). More recently, we have used this system to study the compositions of affinity isolated L1 RNPs (Taylor *et al.*, 2016)–including the interactomic consequences of treating them with a mixture of RNases A and T1 while immobilized on affinity medium, revealing which proteins become labile upon the treatment compared to a control (Taylor *et al.*, 2013 and 2018). The following protocol, detailed fromTaylor *et al.* (2018), will enable users to obtain affinity enriched L1 RNPs suitable for the aforementioned RNase-sensitivity assay and can be easily adapted to include treatments with various enzymes not specifically described herein. A schematic diagram of the procedure is presented in Figure 1.

To execute this protocol the user requires cryomilled, ORF2p-3xFLAG-tagged, LINE-1-expressing cells. HEK-293T_LD_ cells and inducible tagged L1-expression plasmids may be obtained by request. With respect to the final readout of the below described procedure, RNase sensitivity can be assessed by numerous methods (Taylor *et al.*, 2013 and 2018). Among them, SDS-PAGE and general protein staining provide a way to broadly observe visibly changing bands in gels, and this approach is most useful if the identities of proteins within the bands are known (*e.g.*, labeled entities in Figure 4A). Western blotting provides a targeted and more sensitive way to assess the same, however, validated antibodies against the specific target(s) are required. In contrast, differential quantitative mass spectrometry (MS) provides a sensitive and hypothesis-free mode of analysis (*e.g.*, as in Figure 4D). Quantitative MS for these purposes may be conducted using a wide variety of methods, including the use of metabolic labeling, such as Stable Isotope Labeling by Amino acids in Cell culture (SILAC; Ong *et al.*, 2002), described below, or label-free approaches (such as those reviewed in Armean *et al.*, 2013).

## Materials and Reagents

*Note: Catalog numbers for specific suppliers we use are given for many of the reagents listed below; an equivalent quality reagent from an alternative supplier can typically be substituted with comparable results. If a supplier is not explicitly stated, we do not presume any particular source to be superior. Standard materials and reagents for mammalian cell culture are required and are not all explicitly listed below. For experimental procedures not related to cell culture (for which cell culture grade reagents should be used) molecular biology grade is suitable, unless an alternative is explicitly stated. LC-MS grade reagents are recommended for use in mass spectrometry sample workup. The protocols outlined involve work with liquid nitrogen; appropriate clothing and safety precautions are advised. The NuPAGE denaturing electrophoresis system (Thermo Fisher Scientific) is used in this protocol, but nearly any discontinuous or gradient, denaturing protein SDS-PAGE system will do. Simple solutions are described in this section; solutions containing multiple solutes are defined in the Recipes section, at bottom.*

- Cell culture (Procedure A)
  1. Dialysis Tubing, 3,500 MWCO (Thermo Fisher Scientific, catalog number: 68035)
  2. 50 ml polypropylene conical tubes (Thermo Fisher Scientific, catalog number: 339652)
  3. 16 Ga needle
  4. Luer-lock syringes (various sizes, 10-30 ml)
  5. Syringe end caps (Bio-Rad Laboratories, catalog number: 7311660EDU)
  6. Styrofoam box
  7. Parafilm (Millipore Sigma, catalog number: P7793)
  8. Serological pipettes (*e.g.*, from 1 ml to 50 ml)
  9. Gloves
  10. pLD401 or other LINE-1 expression plasmid
  11. Freestyle 293 Medium (Thermo Fisher Scientific, w/o lysine and arginine–custom order)
  12. L-glutamine (200 mM; Thermo Fisher Scientific, catalog number: 25030081)
  13. L-proline
  14. L-arginine
  15. L-lysine
  16. ^13^C_6_^15^N_4_-L-arginine (Arg10; Cambridge Isotope Laboratories, catalog number: CNLM-539-H-PK)
  17. ^13^C_6_^15^N_2_-L-lysine (Lys8; Cambridge Isotope Laboratories, catalog number: CNLM-291-H-PK)
  18. Fetal bovine serum (FBS), tetracycline-free
*Note: Numerous suppliers can provide this. Many suppliers carry FBS products not labeled as tetracycline-free, but consulting the product specification sheet for a given lot may reveal that tetracycline has been tested for and found to be absent or below a ‘negative’ threshold (ideally in the single digit ng/ml range). Check with the supplier for the testing threshold and request a certificate of analysis for the lot to be purchased. Test a sample from that specific lot in case of doubt. In our hands, the performance of such lots has been identical to ‘certified’ tetracycline-free FBS.*
  19. Hybridoma SFM medium (Thermo Fisher Scientific, catalog number: 12045084)
  20. Phosphate-buffered saline (PBS), pH 7.4 (Thermo Fisher Scientific, catalog number: 10010023)
  21. PEI (polyethylenimine) Max (MW 40,000; Polysciences, catalog number: 24765-1)
*
Note: Two grams is enough to transfect > 600 L. To prepare 1 mg/ml PEI Max working solution: dissolve 100 mg PEI Max in 90 ml ddH_2_O, adjust pH to 7.0 using 1 M NaOH, adjust the volume to 100 ml, filter sterilize, and store at 4 °C. Never freeze PEI working solution; working solution can be used for up to 6 months if stored at 4 °C.
*
  22. Doxycycline (Millipore Sigma, catalog number: D9891)
*
Note: Prepare a stock solution of 10 mg/ml in de-ionized water, sterile filter, and store at -20 °C, protected from light. Antibiotic stock solutions may lose activity over time; make them fresh at least every six months, but preferably every three (Okerman et al., 2007). Thaw each stock only once; it may be stored a few days at 4 °C after thawing, and then should be discarded.
*
  23. Liquid nitrogen (LN_2_) and Dewar flask
  24. Heavy SILAC medium (see Recipes)
  25. Light SILAC medium (see Recipes)
- Proteomics (Procedures B, C, and D)
*
Note: For spectrophotometric protein concentration assay, such as the BCA or Bradford assay (Olson and Markwell, 2007), many ready-made kit products for such assays are available.
*
  1. 1.5 ml microcentrifuge tubes (Eppendorf, catalog number: 022363204)
  2. 2 ml safe-lock microcentrifuge tubes (Eppendorf, catalog number: 022363344)
  3. 5 ml or 15 ml tubes
*Note: See Step B3e iv for additional considerations regarding microcentrifuge tubes.*
  4. OMIX C18 pipette tips, 10-100 μl (Agilent, catalog number: A57003100)
  5. Low binding tubes (*e.g.*, Sorenson, catalog numbers: 11300, 39640T); Eppendorf Protein LoBind, Eppendorf Safe-lock (*e.g.*, Eppendorf, catalog numbers: 145525, 145530)
  6. 2-[4-(2-hydroxyethyl)piperazin-1-yl]ethanesulfonic acid (HEPES)
  7. Sodium chloride (NaCl)
  8. Sodium hydroxide (NaOH)
  9. NaH_2_PO_4_·H_2_O
  10. Na_2_HPO_4_
  11. Triton X-100
  12. Protease inhibitor cocktail, EDTA-free, Roche (Millipore Sigma, catalog number: 11873580001)
  13. Ammonium sulfate ((NH_4_)_2_SO_4_)
  14. Mono- and di-sodium phosphate
  15. Coomassie Brilliant Blue G (Millipore Sigma, catalog number: B0770)
  16. Phosphoric acid
  17. Methanol
  18. Hydrochloric acid (HCl; Fisher Scientific, catalog number: A466)
  19. Water (LC-MS grade; Fisher Scientific, catalog number: W6500)
*Note: Prepare solutions used for mass spectrometry sample workup and analyses with LC-MS grade water and the highest purity reagents available.*
  20. Acetonitrile (LC-MS grade; Thermo Fisher Scientific, catalog number: 51101)
  21. Ammonium Bicarbonate (use highest purity grade available; *e.g.*, Millipore Sigma, catalog number: 022363344)
*Note: Prepare 50 ml of a 100 mM solution with LC-MS grade water, filter through a sterile 0.22 µm syringe filter, and store at 4 °C in a clean polypropylene tube. Make fresh every couple weeks.*
  22. Trypsin Gold, Mass Spectrometry Grade (Promega, catalog number: V5280)
*Note: Prepare trypsin stock solution at 100 ng/µl in 1 mM HCl; store in aliquots of up to 20-50 µl at -80 °C.*
  23. Trifluoroacetic Acid (TFA) (sequencing grade, or better; *e.g.*, Thermo Fisher Scientific, catalog number: 28902)
*Note: Produce a 20% w/v TFA stock with LC-MS grade water.*
  24. Acetic Acid (Fisher Scientific, catalog number: 60-046-912)
  25. (Optional) Antibodies for Western blotting
*Note: Commercially available antibodies that we have used with success to identify ORF1p and ORF2p-3xFLAG by Western blotting.*
    - α-ORF1p, clone 4H1 (Millipore Sigma, catalog number: MABC1152), primary antibody, titer–1:5,000 (original concentration 2 mg/ml; final concentration 0.4 µg/ml)
    - Anti-FLAG M2 antibody (Millipore Sigma, catalog number: F3165 or F1804), primary antibody, titer–1:2,000 (original concentration 1 mg/ml; final concentration 0.5 µg/ml)
    - α-mouse IgG, HRP-linked whole antibody (GE Healthcare, catalog number: NA931-1ML), secondary antibody for enhanced chemiluminescence, titer–1:10,000.
  26. Anti-FLAG M2 antibody (Millipore Sigma, catalog number: F3165 or F1804)
  27. Dynabeads M-270 epoxy (Thermo Fisher Scientific, catalog number: 14302D)
  28. Glycerol
  29. Bovine Serum Albumin (BSA; Standard Ampules, 2 mg/ml, Thermo Fisher Scientific, catalog number: 23209)
  30. 1 M iodoacetamide stock solution
*Note: Best practice is to prepare iodoacetamide in water, fresh; iodoacetamide is light sensitive and chemically labile. In our experience, 1 M aliquots can be stored at -20 °C, protected from light, and are still functional for several months.*
  31. RNase A/T1 Mix (Thermo Fisher Scientific, catalog number: EN0551)
  32. 4x LDS (lithium dodecyl sulfate) sample loading buffer (Thermo Fisher Scientific, catalog number: NP0007)
  33. 10x sample reducing agent (Thermo Fisher Scientific, catalog number: NP0004); or 500 mM dithiothreitol (DTT)
  34. 4-12% Bis-Tris gels, 10-well, 1 mm (Thermo Fisher Scientific, catalog number: NP0321BOX)
*Note: Sample volumes loaded as described here may be up to 60-70 µl in volume, exceeding the standard well capacity. Thus, each sample is loaded twice (described in Step D4). After the first loading the sample is run just into the gel, and then the remainder is loaded into the same well and the run is completed. Alternatively, gels with sufficient well capacities for a single loading may be used, such as Bolt 4-12% Bis-Tris Plus Gels, 10-well, 1 mm (Thermo Fisher Scientific, catalog number: NPNW04120BOX).*
  35. 20x MOPS (3-morpholinopropane-1-sulfonic acid) gel running buffer (Thermo Fisher Scientific, catalog number: NP0001)
  36. 10x PBS stock solution (see Recipes)
  37. 0.1 M sodium phosphate pH 7.4 (see Recipes)
  38. 3 M Ammonium sulfate (see Recipes)
  39. Magnetic Media Storage Solution (see Recipes)
  40. Protein extraction solution (see Recipes)
  41. Gel acidification solution (see Recipes)
  42. Blue Silver stain (see Recipes)
  43. Gel plug destaining solution (see Recipes)
  44. Tip wetting solution (see Recipes)
  45. Tip wash solution (see Recipes)
  46. Elution 1 solution (see Recipes)
  47. Elution 2 solution (see Recipes)

## Equipment

*Note: Typical molecular biology lab equipment is required, not all explicitly listed below. catalog numbers are given for most of the equipment as examples; instruments from alternative manufacturers may be substituted provided equivalent functionality. Equipment has been listed approximately in order of use.*

- Cell culture (Procedure A)
  1. Pipettes
  2. CO_2_ incubator for mammalian cell culture
  3. Scalpels (*e.g.*, size 11) or razor blades
  4. New Brunswick Innova 2000 platform shaker (Eppendorf, catalog number: M1190-0002; or Thermo Fisher Scientific, model: Forma Model 416; or equivalent)
*Note: Any shaker installed in a mammalian cell culture incubator must be able to tolerate continuous high humidity (~90% relative humidity).*
  5. Swing-bucket centrifuge rotor
  6. 1 L square PYREX glass bottles (Corning, catalog number: 1396-1L)
*Note: Plastic bottles inhibit transfection (polypropylene, polyethylene, PETG)*
  7. Erlenmeyer flasks
  8. Ice bucket
  9. Hemocytometer
  10. High speed centrifuge with appropriate rotor for pelleting cells from large volumes (*e.g.*, for ~250 or 500 ml bottles)
- Proteomics (Procedures B, C, and D)
  1. Metal spatula
  2. Milling balls, stainless steel, 20 mm (Retsch, catalog number: 05.368.0062)
  3. 50 ml stainless steel milling jar (Retsch, catalog number: 01.462.0149)
  4. PTFE milling jar insulator (custom made, see LaCava *et al.*, 2016)
*Note: The use of a jar insulator is optional, but strongly recommended.*
  5. Planetary Ball Mill PM 100 (Retsch, model: PM 100, catalog number: 20.540.0001)
  6. Vacuum centrifugal concentrator (a.k.a. speed-vac)
  7. Neodymium magnet microfuge tube rack (Thermo Fisher Scientific, catalog number: 12321D)
  8. Thermomixer (Eppendorf, catalog number: 5355000.011; or equivalent)
  9. Refrigerated microcentrifuge (capable of reaching ~20,000 RCF)
  10. Microtip sonicator (*e.g.*, Qsonica, model: Q700) equipped with a low intensity 1/16” microtip probe (Qsonica, catalog number: 4717)
  11. SDS-PAGE electrophoresis system (*e.g.*, Thermo Fisher Scientific NuPage)
  12. Gel knife or chisel
  13. Orbital shaker
  14. Low-extractable borosilicate glass vials with chemically resistant caps (*e.g.*, Kimble, catalog number: 74515-20)
*Note: These are recommended for the storage of stock and working solutions used for MS.*
  15. Quadrupole-Orbitrap mass spectrometer (Thermo Fisher Scientific Q Exactive or Fusion series) interfaced with a nano-flow liquid chromatography (LC) system (*e.g.*, Thermo Scientific EASY-nLC 1200 or UltiMate 3000 RSLCnano)
*Note: Older model LC-MS systems may also be perfectly suitable; at the time this analysis was initiated we were using LTQ-Orbitrap models, see Step D7.*

## Software

1. MaxQuant
2. (Optional) R

## Procedure

The protocol below anticipates that suspension-grown, SILAC-labeled cells will be used to enable a final readout by quantitative MS. In the case that SILAC labeling is not necessary for your experimental design, effective standard procedures for L1 expression in HEK-293T_LD_ cells are thoroughly described in Taylor *et al.*, 2016; review of this reference is recommended in any case. In the case that adherent cells will be SILAC labeled, see Taylor *et al.*, 2013.

- Express ORF2p-3xFLAG-tagged L1 in SILAC labeled HEK-293T_LD_ suspension cultures
SILAC-labeled cells will be transfected in suspension using pLD401 plasmid DNA, and/or other 3xFLAG-tagged constructs such as pMT302 (Taylor *et al.*, 2013), and PEI (polyethylenimine); after transfection, L1 expression will be induced by the addition of doxycycline (Dox; typically 1 μg/ml). High-quality endotoxin-free DNA is critical to success. pLD401 contains a synthetic, recoded human L1 sequence (*Orfeus*-Hs) encoding a carboxy-terminal 3xFLAG-tagged ORF2p, expressed under the control of the tetracycline response element promoter (An *et al.*, 2011; Taylor *et al.*, 2013). Suspension culture yields large human cell pellets with minimal work, waste, and cost when compared to monolayer adherent growth. We typically obtain ~1.5 g HEK-293T_LD_ cells, wet cell weight, per 100 ml culture. In the past we have used square Corning Pyrex bottles in an orbital platform shaker at 130 rpm, installed inside of a 37 °C, humidified incubator, maintained at 5%-8% CO_2_ (Muller *et al.*, 2005; Taylor *et al.*, 2016; Domanski and LaCava, 2017); however, more recently we have obtained comparable results using Erlenmeyer flasks at the same speed. For instructions on setting up the hardware for such a system, refer to Taylor *et al.*, 2016. In addition to procedures we describe, standard mammalian cell culturing techniques and best practices apply (Freshney, 2011; Uphoff and Drexler, 2013).
If starting from adherent HEK-293T_LD_ cells, follow the HEK cell suspension adaptation protocol presented in Domanski and LaCava, 2017; however, unlike the cells described in that procedure, frozen stocks made from suspension-adapted HEK-293T_LD_ cells can be used to directly inoculate new suspension cultures successfully. If starting from a frozen stock or recently suspension-conditioned HEK-293T_LD_ cells, proceed as detailed below. Cells may grow to a density of ~10 million/ml but growth slows after ~5 million/ml and transfection efficiency drops when cells are not in logarithmic growth.
***IMPORTANT:*** *We have observed that, over time, the Dox-inducibility of HEK-293T_LD_ can markedly decline. This has been determined to be caused by loss of expression of rtTA. Therefore, these cells should be periodically re-selected for blasticidin resistance (the BSD gene [Kimura et al., 1994]) was co-incorporated with rtTA), ensuring a transcriptionally active rtTA locus. Although keeping cells under continuous selection is the surest way to ensure a maximally Dox-responsive cell population, cells selected prior to making frozen stocks respond in sufficient proportion and uniformity for the work described below; it is also gentler on the cells overall. We therefore periodically re-select HEK-293T_LD_ cell populations under challenge of 10 µg/ml blasticidin S prior making stocks, but do not maintain cells under continuous selection.
*
***Practical note:*** *Before proceeding to full-scale expression in SILAC medium, we recommend optimization experiments in standard medium to verify transfection efficiency and Dox induction response within your setup and implementation. We recommend parallel transfections with a Tet-On GFP plasmid and a constitutively active GFP plasmid, with DNA prepared at the same time and in the same method as the pLD401 plasmid to be transfected. The ratio of PEI-max to DNA may be varied along with other parameters, see Longo et al., 2013; Stuible et al., 2018. Prior to culturing cells in suspension at full-scale, it may also be helpful to compare the per-cell expression of ORF1p and/or ORF2p to that of cells grown in adherent cultures, transiently transfected with either PEI-max or a commercial cationic lipid-based reagent, maintaining other variables comparably to suspension culture. Proper suspension culture produces indistinguishable per-cell expression from adherent culture transfected with either method (see Taylor et al., 2018, Appendix 1–Figure 1).
*
  1. Prepare heavy and light SILAC media (see Recipes).
    - Dialyze Tet-free FBS to remove amino acids for 24 h, at 4 °C, against 10-volumes of PBS with 2 buffer changes using 3500 MWCO dialysis tubing.
*Example: 500 ml FBS, dialyzed against 5 L PBS starting at ~noon (Day 1). Before leaving the lab in the evening change the buffer (5 L fresh PBS). First thing the next morning (Day 2) change the buffer again; retrieve dialyzed FBS ~noon. For this regime, it is helpful to have two 10 L buckets.*
    - For ‘heavy’ SILAC medium use Arg10 and Lys8; for ‘light’ SILAC medium instead use naturally occurring L-arginine and L-lysine.
    - Sterilize the media by filtration.
  2. Inoculate otherwise identical heavy and light SILAC suspension cultures at a cell density of ~0.2 million cells per ml in 200 ml.
  3. Grow to ~5 million/ml.
Obtaining accurate cell counts can be challenging because HEK-293T_LD_ tends to grow in small clumps of up to ~30 cells in suspension. Accurate counting requires dissociation of the clumps by gentle trituration using a pipette.
    - Using a 1 ml Serological pipette, aliquot 200 μl of culture to a clean microcentrifuge tube.
    - Mix by flicking, and pipette 10 μl onto one side of the hemocytometer (also called hemacytometer).
    - With a 200 μl pipette, set the volume to ~150 μl and triturate 30 times to break up clumps. Try not to foam.
    - Pipette 10 μl onto the remaining half of the hemocytometer.
    - Visualize the cells before and after cell clump dissociation by trituration. Shearing during trituration may lyse a small fraction of the cells. Increase or decrease trituration if appropriate to get accurate cell counts with minimal lysis. Properly triturated samples can also be counted in automated cell counters and leverage vital stains.
  4. Passage these cells, which have now doubled ~5 times, inoculating into fresh heavy and light SILAC suspension cultures at a cell density of ~0.2 million/ml in 200 ml.
***IMPORTANT:*** *From the time of seeding cells in SILAC suspension, until the time of harvesting, the cells should have been permitted to double ≥ 10 times to assure thorough Arg10/Lys8 metabolic labeling of the proteome. Following the scheme presented here starting from Step A2, above, through Step A8, below, assures 11 doublings. Additional passages prior to induction, if convenient, provides similar results.*
  5. Grow to ~2.5 million/ml in 200 ml medium (~4 doublings). We have successfully transfected at up to 4 million/ml but recommend this concentration to ensure logarithmic growth.
  6. On **Day 1**–transfect the cells in suspension
Each 200 ml suspension culture will receive 1 µg/ml pLD401 DNA and 3 µg/ml PEI ‘Max’ 40 kDa, prepared in ~1/20^th^ the final culture volume hybridoma SFM medium. A master transfection mix for the two cultures is made. The following example assumes 2 x 200 ml SILAC cultures–one heavy, one light–and transfection with pLD401 (ORF2p-3xFLAG). If working with a larger number, scale up accordingly. A parallel GFP plasmid may be used as a control for transfection efficiency, we recommend a Tet-On plasmid (in which case, add 1 µg/ml Dox to the control GFP culture at the time of transfection).
    - Warm 20 ml hybridoma-SFM medium to room temperature, 10 ml hybridoma-SFM per 200 ml culture to be transfected.
    - Add 400 μg pLD401 to the hybridoma-SFM and mix well, *e.g.*, by vortexing.
    - Add 1.2 ml of 1 mg/ml PEI Max (pH 7.0) to the hybridoma-SFM/DNA solution and mix well.
      Final volume should be ~22-23 ml.
    - Incubate for 15 min at room temperature to allow DNA-PEI complexes to form.
    - Pipette half of the transfection mixture into each 200 ml culture and return to the incubator.
  7. On **Day 2**–split cells 1:2.5-1:3
    Transfer half (100 ml) of each transfected SILAC suspension culture to each of two 1 L bottles containing 150-200 ml of fresh SILAC medium, resulting in 2 x 250 ml or 2 x 300 ml cultures for both heavy and light.
*Note: If desired, before the decision to split the cells and use more SILAC media, check the efficiency of the GFP control transfection culture.*
  8. On **Day 3**–induce each culture by addition of dox at a final concentration of 1 μg/ml.
The cell density at the time of induction should be ~4 million/ml (~2 doublings, constituting ~11 total doublings to this point). Dilute accordingly or wait an additional day if needed. L1 expression should be induced for ~24 h, by 48 h, ORF2p expression declines (Taylor *et al.*, 2013); shorter induction times may be sufficient but should be verified.
  9. On **Day 4**–harvest cells by centrifugation and extrude into liquid nitrogen.
    - Count cells; counts are useful for normalization and blotting.
    - Gather an ice bucket and clean centrifuge bottles.
    - Transfer a 1 ml aliquot(s) of each culture for Western blotting to a microcentrifuge tube. Spin at ≤ 1,000 RCF for 30 s, aspirate the media, and store on ice until freezing is convenient.
***IMPORTANT:*** *Check for comparable levels of ORF1p and ORF2p-3xFLAG expression in heavy and light labeled cell cultures by Western blot (antibodies and titers described in Materials and Reagents* *B25). If the expression levels are very different (greater than* ±*20%), the samples may not be suitable for quantitative comparisons or will require thoughtful normalizations.*
    - Spin the cultures at ≤ 1,000 *× g* for 10 min at 4 °C to pellet.
      This can be done in several 50 ml conical tubes for each batch, or in fewer, larger bottles.
*Note: The pellets should appear approximately uniform. If centrifuged too hard, cells will be crushed, forming two different-colored layers. The goal here, and in subsequent centrifugations of these cells, is to obtain a relatively tight-packed cell pellet that maximally excludes buffer without breaking the cells. We consider 1,000 × g to be the maximum nominal centrifugal force to be used. We are commonly using a Sorvall T6000D or Beckman Allegra X-14R with swing-bucket centrifuge rotor at 2,000 rpm (~830-930 x g). One may want to establish their choice operating speeds in a preliminary experiment.*
  10. Resuspend the pellets in a minimal volume of PBS (approximately equal volume to the pellet) using a 25 ml pipette set to ‘slow.’ One may pool resuspended cells.
*Note: Additional washes may be added if needed; however, we attempt to limit the amount of handling before freezing. Moreover, one may choose to handle the cells obtained from each suspension culture separately, e.g., in case of potential differences in metabolic labeling efficiency (see below), transfection, or Dox induction efficiency between cultures. Powders produced from similar cell populations may be mixed after milling and quality control (Procedure B) if desired; when kept separate, each culture represents a biological replicate. On the other hand, pooling is often advantageous for sample handling and may be done at this point.*
  11. Pellet cells inside syringes.
    - Select syringe(s) for cell pelleting. Syringes with capacities in the range of 10-30 ml are typically appropriate for suspension culture yields.
    - Remove the plungers and set aside.
    - Securely cap syringes with Luer-lock end caps at spout, Parafilm at plunger opening, and place inside 50 ml conical tubes.
    - Spin at ≤ 1,000 *× g* for 10 min at 4 °C. Transfer syringes to an ice bucket.
      - During the centrifugation, insert a conical tube rack in a Styrofoam box. Fill the box with LN_2_ to at least the mid-point of the rack.
      - Pre-label 50 ml conical tubes. Punch a number of holes in the caps of the tubes using a 16 Ga needle. This will permit the LN_2_ to be drained from the tubes later without loss of cell material. These caps will later be replaced with un-punched caps. To avoid plastic waste, punch caps taken from used 50 ml conical tubes destined for disposal (wash caps first) and save punched caps for future use. Each tube will hold approximately 15-20 g extruded cells, which we often refer to as ‘BBs’ because of their round shape.
      - Transfer the pre-labeled tubes to the rack in LN_2_. Fill the tubes with LN_2_.
    - Aspirate the PBS, leaving only the wet cell pellet in the syringe.
  12. Extrude the cells into the LN_2_ as follows. Remove the Luer lock cap. Positioning the bottom of the syringe over the 50 ml conical tube with LN_2_, insert the syringe plunger carefully. Extrude the cells gradually into the tube containing LN_2_. If injected too fast, they will form large clumps. If more LN_2_ is needed, carefully pour more in. Use the pre-chilled spatula as needed to break apart any clumps.
  13. Wearing appropriate protective gloves, cap the tube using the punched lid. Pressure may build up and cause LN_2_ to spurt out through the punches. Exercise caution.
  14. Decant LN_2_ into dewar or Styrofoam box and replace the punched lid with an intact lid. Store the tubes at -80 °C until cryomilling.
- Cryomill L1-expressing HEK-293T_LD_ cells and conduct quality control checks
  1. Cryogenically disrupt the cell pellets
Cryo-milling L1 expressing HEK-293T_LD_ cells has been previously described in intricate detail, see LaCava *et al.*, 2016; Taylor *et al.*, 2016. Briefly:
    - Pre-cool an insulator, milling jar, 2 x 20 mm grinding balls, a metal spatula, and a 50 ml tube by immersing in LN_2_.
    - Place the cell pellets inside the jar, jar inside the insulator, and assembly inside the Retsch PM 100 milling apparatus.
    - Set the counter balance and clamp the jar in place. Run the machine for 3 milling cycles of 3 min each (reverse rotation, 1 min interval, no break time) at 400 rpm.
    - Cool down the milling jar with LN_2_ between cycles.
    - Recover the resulting cell powder using a spatula and transfer to 50 ml tube. Store at -80 °C.
  2. Assess the comparability of the cell powders by protein content
Extract 25-50 mg of each cell powder into 9 parts (225-450 µl) protein extraction solution (see immediately below) and check by protein assay that each yields a similar protein content (typically ±10%). Extracts may be produced more dilute if greater volume is advantageous. Save 10 µl for checking heavy amino acid incorporation (Step B3, below). The procedure essentially follows the rationale laid out in Shevchenko *et al.*, 2006.
    - Combine cell powder with your favorite protein extraction solution (*e.g.*, what you may use for immunoprecipitation–see Recipes), vortex (and if necessary, briefly sonicate as per Step C2f) to homogenize, and then centrifuge the extract for 10 min at 20,000 *× g* to produce a clarified extract that can be measured by *e.g.*, Bradford or BCA assays.
    - Alternatively, extract proteins in a Tris-buffered solution containing 2% (w/v) SDS and measure protein content by BCA assay–SDS is not well tolerated by the Bradford assay (Zor and Selinger, 1996). SDS-based solutions tend to produce viscous cell extracts requiring a greater degree of sonication than typical IP solutions.
***IMPORTANT:*** *If comparable protein quantities are obtained from similar masses of cell powder, future experiments and comparisons may be calibrated to the mass of powder extracted. If the respective yields of protein from heavy and light SILAC cell powders made under identical extraction conditions are not comparable, then one source of material may have retained more liquid in the cell pellet before extrusion into LN_2_ (i.e., incomplete PBS removal) or may have accumulated excessive condensate due to storage at -80 °C with an improperly closed cap. Additional water weight will reduce the apparent yield of protein per mg of cell powder. In this case, future IP experiments using those materials should be calibrated by relative protein content per mass of powder.
*
  3. Assess the percentage of heavy amino acid incorporation.
***IMPORTANT:*** *In order to make quantitative comparisons by mass spectrometry using SILAC methods, heavy SILAC labeling should be thorough, ~95% or better. Provided (a) the heavy SILAC proteome is thoroughly labeled with Arg10 and Lys8, and both (b) target protein expression, as assessed by Western blotting (done in Step A9c, above), as well as (c) protein content per mass of cell powder, as assessed by e.g., a colorimetric assay (Step B2, above), are both in good agreement between heavy and light materials, then these materials are ideal for quantitative mass spectrometry work*.
    - Conduct SDS-PAGE of clarified cell extracts from heavy-labeled cells.
      - Add DTT to 25 mM to the 10 µl sample(s) (Step B2a) and heat at 70 °C for 10 min, and then briefly chill samples on ice.
      - Add iodoacetamide to 100 mM and incubate at room temperature for 30 min in the dark.
      - Load a range of aliquots–*e.g.*, 1, 2, and 4 µl or 3 and 6 µl–on *e.g.*, a 10-well, 1 mm, 4-12% Bis-Tris gel.
*Notes:*
        1. *Loading a range ensures the likely production of gel plugs with an appropriate quantity of protein, despite variations in extract efficiency, etc.*
        2. *Typical liquid chromatography columns attached to mass spectrometry systems have loading capacities of ~1 µg. In our hands, loading the peptides ultimately obtained from a 4 µl aliquot of extract and following the handling procedures described here, only approaches ≤ 20% of the column capacity upon loading. This is sufficient to estimate the H-label incorporation. More material may be loaded at the user’s discretion.*
      - Electrophorese the samples until they migrate ~6 mm into the gel as assessed by gel loading dye at the sample migration front.
    - Fix and Coomassie stain the gel.
      - We use ‘blue silver’ (see Recipes), with the following modified pre-washing procedure for NuPAGE Bis-Tris gels. All steps that follow are carried out at room temperature until otherwise stated.
        1. After opening the gel cassette, with the gel adhered to one of the plates, use a clean gel knife/chisel to trim away the wells and the portion of the gel below the 6 mm mark (readily discerned by the position of the loading-dye front).
        2. Transfer the upper portion of the gel from the plastic cassette to a clean plastic 15 cm diameter petri dish and incubate for 10 min in 40 ml of de-ionized water with gentle agitation on an orbital shaker.
        3. Remove the water and replace it with 40 ml of 0.1 M HCl; incubate for 10 min.
        4. Remove the 0.1 M HCl and replace it with 40 ml of water; incubate for 10 min.
        5. Remove the water and replace it with 40 ml of blue silver stain; incubate for 2 h to overnight. If the gel has been properly washed **in the prior 3 steps**, the blue silver will remain a deep, translucent green color due to maintenance of the low pH dye solution. If the washing is incomplete, residual buffering capacity within the gel may turn the stain blue-ish, and/or, the gel may develop uneven background staining. Changing the stain and incubating several hours to overnight in fresh (green-hued) blue silver will typically resolve this problem and result in mild, uniform background staining with major bands clearly visible above background.
      - Destain background thoroughly–several hours to overnight.
        1. For blue silver, simply use several changes of de-ionized water.
        2. You may choose to take an image of the gel. Always clean surfaces used to image gels that will contribute sample to mass spectrometry analyses.
    - Cut the regions of gel containing the samples from just below the well to just above the dye front (referred to as a ‘gel plug;’ will appear similar to those depicted in Figure 4C, but more protein rich)–**Day 1**.
*Note: If extracted 1:9 as suggested in Step B2a, the 2 or 4 µl samples should contain a sufficient quantity of protein for this analysis. The gel should be cut upon a clean surface. We use a rectangular glass plate placed over white paper, which aids visibility of the gel and its pieces. Solutions used below should be prepared with ‘mass spectrometry’ grade reagents.*
      - Using a clean razor blade or scalpel, chop each one into 1 mm^3 ^cubes (about 25 pieces per gel plug), and transfer to a 1.5 ml microfuge tube.
*Note: The blade may be cleaned of residual gel fragments when necessary by agitating it in a beaker of molecular biology grade water; residual liquid can be wicked away by touching the tip of the blade to a clean low-lint tissue (‘Kimwipe’).*
      - Add 500 µl of ‘gel plug destaining solution’ to the gel pieces in the tubes (50 mM ammonium bicarbonate in 50% (v/v) acetonitrile; see Recipes).
      - Mix with the gel pieces by vortexing.
*Note: If the color of the solution changes to dark blue immediately, discard it and add another 500 µl of destaining solution. One milliliter may be used here instead of 500 µl; repeat multiple times if the solution becomes blue quickly.*
      - Incubate at 37 °C for 1 h with shaking in a Thermomixer (1,000-1,200 rpm); discard the supernatant. The gel pieces should be in motion, not settled at the bottom of the tube.
      - Add another 500 µl of destaining solution if the gel pieces are still blue.
*Note: Continue to destain as needed (this step can go overnight).*
      - Remove destain when the gel pieces no longer exhibit blue staining.
*Note: They should appear whitish and semi-transparent with no obvious blue remaining.*
      - Dehydrate gel pieces with 500 µl acetonitrile, vortex, let the tubes sit at room temperature for 2 min or more.
*Note: Gel pieces will appear opaque and thoroughly white.*
      - Remove acetonitrile from all samples.
      - Dry samples in a vacuum centrifuge (‘speed-vac’) for 10 min.
      - Samples can be stored at -20 °C or proceed to trypsin digest.
    - In-gel digestions of proteins to peptides using trypsin–**Day 2**.
      - Prepare all tubes on ice.
      - Prepare trypsin working solution: 12.5 ng/µl in 50 mM ammonium bicarbonate. Our trypsin stock is 100 ng/µl in 1 mM HCl.
      - Add enough 12.5 ng/µl trypsin to cover the gel pieces.
*Note: This will vary by gel plug dimensions, but in our experience is ~38 µl for gel plugs obtained from 15-well, 1 mm thickness NuPAGE gel, and ~90 µl for a 10-well gel of the same*.
      - Let samples swell for 45 min on ice.
      - After swelling, add 50 mM ammonium bicarbonate to cover gel pieces if necessary.
*Note: Typically, ~12 µl for 15-well or ~30 µl for 10-well, 1 mm thickness gel plug.*
      - Move the tubes to a 37 °C incubator to digest overnight (or at least 6 h).
    - Terminate the digest; collect and pool the peptides–**Day 3**.
      - Produce aliquots of 2% and 0.1% (w/v) TFA from the 20% stock.
      - Add 1/3^rd^ the digestion volume of 2% (w/v) TFA to the digestion mix (0.5% w/v TFA final concentration) to stop digestion.
      - Incubate at room temperature for 5 min with shaking in a Thermomixer (1,000-1,200 rpm) or vortex mixer equipped with multi-tube holder (setting 2-3 on a vortexer with a max intensity of 10).
      - Remove the acidified digest solutions to microcentrifuge tubes (‘protein low-binding’ optional); hold at room temperature or 4 °C while pieces are further extracted.
***IMPORTANT:*** *Performance may vary, some considerations discussed here (Kraut et al., 2009). Tubes that will be exposed to 40%+ acetonitrile (as below) may benefit from being pre-washed with pure acetonitrile. Some tubes may leak plasticizers or unincorporated (co)polymers when challenged with high concentrations of organic solvents. Some examples of polymer contaminated mass spectra are displayed in LaCava et al., 2015, Figure 5. The best practice is to empirically test your tubes, and then repeat these tests periodically (or, when a problem occurs) because occasional (usually unannounced) manufacturing changes may alter performance. Pre-washing reduces the potential for contamination of the sample by components of tube manufacturing by first solubilizing them in e.g., high acetonitrile and removing them with the wash. We routinely use ~100 µl acetonitrile to wash 0.65 ml tubes and ~300 µl to wash 1.5 ml tubes as a precaution (although it may not always be necessary–testing is advised for any doubt). Tubes we commonly use: Sorenson low binding (e.g., #11300, #39640T), Eppendorf Protein LoBind, Eppendorf Safe-lock, Research Products International (e.g., #145525, #145530). Importantly, the same rules apply for plastic pipette tips used for handling solvents! Use only high quality, solvent resistant tips. These are also available in low protein binding varieties if desired.
*
      - Extract with 50 µl of 0.1% (w/v) TFA at room temperature for 45 min in a Thermomixer or vortex mixer equipped with multi-tube holder, speed setting as above.
      - Remove the extractants and combine them with the cognate **digest solutions**. The total volume may be ~100 µl for 15-well and ~150 µl for 10-well gel plugs.
    - Desalt peptides using OMIX tips (C18, 100 µl) by centrifugation.
***IMPORTANT:*** *The manufacturer’s protocol stipulates pipette-based desalting. We instead use a centrifugal approach because in our experience loading the samples on top of the C18 bed reduces the possibility of (albeit rare) micro-fragments of polyacrylamide gel (or other debris) remaining in the final desalted fraction–such debris may cause column clogging if applied to the mass spectrometer’s liquid chromatography column. This centrifugal approach is also beneficial when many samples are to be processed in parallel. If working with only few samples (as may be the case if assessing the heavy-label incorporation of only 1 or 2 cell cultures) you may implement the manufacturer’s pipette-based protocol using the solutions described below–but be mindful of potential debris carryover if column clogging issues are observed.*
      - Make the following before starting (solutions modified from manufacturer’s instructions; see Recipes). Calculate the amount of each solution needed based on the number of samples to be processed plus 10% volume.
        1. Make adaptors to hold the OMIX tips on top of the 2 ml collecting tubes (to collect waste) or 1.5 ml low binding tubes (to collect elution) during centrifugation steps. See Figure 2.
          **Figure 2.**
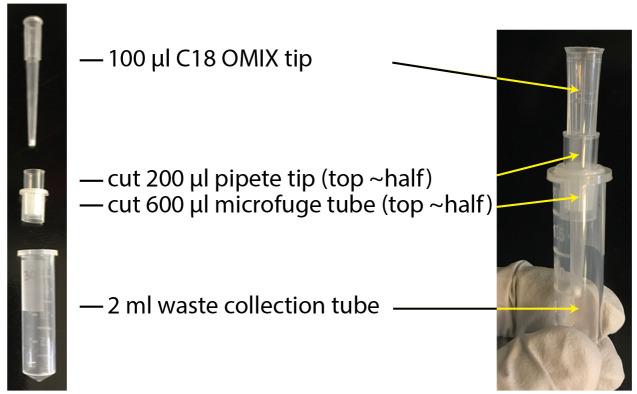
Centrifugal device for solid phase extraction of tryptic peptides for LC-MS/MS. The individual parts are labeled on the left and shown as assembled on the right.
        2. Tip wetting solution: 200 µl per sample.
        3. Tip washing solution: 600 µl per sample.
        4. Elution 1 solution: 80 µl per sample.
        5. Elution 2 solution: 80 µl per sample.
        6. Carefully label each OMIX tip to help keep track of which sample is loaded on which tip during the following centrifugation steps.
        7. Similarly, label a set of clean 1.5 ml low binding tubes (pre-washed with 100% acetonitrile) for collecting eluted peptides.
        8. Put each tip in a 2 ml collecting tube and proceed.
      - Wet the tips by applying 2x 100 µl of tip wetting solution.
        1. Add 100 µl of wetting solution.
        2. Spin at 500 rpm for 1 min (27 *× g* in an Eppendorf centrifuge 5415).
        3. Repeat the two steps above, once.
*Note: Check the volume of waste collected during centrifugation steps; make sure that the OMIX tip will not touch the waste in the collecting tube. Aspirate the liquid in the collecting tubes when needed.*
      - Wash tips by applying 2x 100 µl of tip washing solution.
        1. Add 100 µl of washing solution.
        2. Spin at 500 rpm (27 *× g*) for 1 min.
        3. Repeat the two steps above, once.
      - Bind the samples to C18 resin.
        1. Add the **digest solutions** from each gel plug (≥ 100 µl) to the appropriate OMIX tips. (The tips should accept ≥ 200 µl.)
        2. Spin at 500 (27 *× g*) for 1 min.
        3. Check each tip, make sure that the entire sample passes through the filter.
      - Wash the samples by applying 4x 100 µl of tip washing solution.
        1. Add 100 µl of washing solution.
        2. Spin at 500 rpm (27 *× g*) for 1 min.
        3. Repeat the two steps above, three times.
      - Elute the desalted peptides from the C18 resin.
        1. Transfer the OMIX tips from the 2 ml collecting tubes to the set of 1.5 ml sample collection tubes. (Make sure that the label on the OMIX tip matches the label of the tube.)
        2. Elution #1: apply 80 µl elution 1 solution to the OMIX tips.
        3. Spin at 500 rpm (27 *× g*) for 1 min. (Check that all the solution has passed through.)
        4. Elution #2: apply 80 µl elution 2 solution to the OMIX tips. Elutions #1 and #2 will be collected in the same 1.5 ml tube.
        5. Spin at 500 rpm (27 *× g*) for 1 min. (Check that all the solution has passed through. Refer to the combined fractions as **peptide solutions**.)
      - Put the tubes containing the **peptide solutions** in a vacuum centrifuge and run until dry. (The time varies by vacuum strength.)
    - Liquid Chromatography Tandem Mass Spectrometry (LC-MS/MS): MS methods will vary depending upon the liquid chromatography (LC) setup, the model of MS instrument, and the operator. We provide the following only as guidelines for conducting shotgun proteomic analyses of affinity enriched samples using an Orbitrap-based hybrid mass spectrometer for LC-MS/MS (Gillet *et al.*, 2016). We typically resuspend our dried samples in 10 µl of 0.5% (v/v) acetic acid (or, samples are initially concentrated to this volume). On a first run, ~1/2 or preferably ~1/3 of the sample will be loaded onto the LC column for MS analysis (permitting double the quantity to be used in a second run if needed). The column bound peptides are eluted in an acetonitrile gradient (typically reaching ≥ 40% (v/v) final concentration of acetonitrile over ~1 h), in the presence of an ion pairing reagent such as acetic acid (0.1 M) or formic acid (0.1% v/v) at a flow rate of 200-300 nl/min. Data dependent MS^2^ fragmentation spectra (*e.g.*, of the top 20 most abundant MS^1^ ions) are acquired via collision induced dissociation (CID) or high-energy collisional dissociation (HCD). The resulting mass spectra then require processing in a suitable software package to interpret the proteins present as well as relative quantities and specificities thereof.
*
Note: MaxQuant (Cox and Mann, 2008) is a widely used, freely available MS processing software package with detailed protocols available (Tyanova et al., 2016)–our procedure is summarized below *.
    - Data processing: Use MaxQuant to search the raw data against a database of human protein sequences (we typically us a www.uniprot.org fasta file), as well as a decoy database of reversed protein sequences (set in software), with Arg10 and Lys8 as potential heavy labels. Note that although ORF1p and ORF2p are now represented in Uniprot (entries Q9UN81 and O00370, respectively), you may want or need to add custom sequences for ectopically expressed L1s to your database. For the determination of heavy labeling, we do not typically use the “re-quantify” option; and a typical result with these settings will identify and quantify ≥ 300 proteins. The “peptide.txt” output file can be used to calculate the heavy amino acid incorporation (Geiger *et al.*, 2011). Peptides corresponding to known exogenous contaminants such as keratin and trypsin should be removed. Lysine- and arginine-containing peptides are considered separately. The “ratio H/L” for each peptide is converted to H/(H+L) using the equation H/(H+L) = (“ratio H/L”)/(1 + “ratio H/L”). The median values of H/(H+L) for lysine- and arginine-containing peptides are then obtained. Note, these values underestimate incorporation, as a peptide “ratio H/L” cannot be calculated by MaxQuant when there is no detectable signal at the mass-to-charge ratio of the light partner, as would be expected in the case of full incorporation of heavy amino acid. If the average of the median values of the lysine- and arginine-containing peptides is ~95% or better, it is indicative of thorough heavy labeling and suitability for quantitative comparisons by SILAC.
- Differential affinity immobilization
  1. Production of anti-FLAG magnetic medium. The procedure has been summarized below. We refer the reader to the following references: Cristea and Chait, 2011; Domanski *et al.*, 2012; and Taylor *et al.*, 2016 for comprehensive background on the procedure. Both the Cristea and Chait, and the Taylor *et al.* references contain detailed step-by-step procedures. However, one may also simply follow the manufacturer’s instructions (Thermo Fisher Scientific). Briefly:
    - Prepare 20 μl of 0.5 μg/μl antibody (anti-FLAG M2), in a sodium phosphate (pH 7.4) buffered 1 M ammonium sulfate solution, per mg of magnetic beads to be antibody-conjugated (*i.e.*, 10 μg of antibody, in 20 μl solution, will be used per mg of beads).
*
Note: Millipore Sigma product F1804 contains 50% glycerol and will need to be buffer exchanged prior to epoxy coupling; product F3165 can be used directly (Domanski et al., 2012).
*
    - Combine the antibody solution and the magnetic beads and incubate overnight (16-24 h) at 30-37 °C with rotation. The mixing applied should be sufficient to ensure the beads remain in suspension for the duration of the incubation. For small batches, conjugation can be performed at 37 °C in a thermomixer (≥ 1,200 rpm); however, evaporation and the formation of condensate away from the bulk solution should be avoided, so appropriately even heating or sufficiently thorough mixing are required.
    - After coupling, remove the excess antibody solution, wash the beads to remove residual uncoupled antibodies.
*Note: You may choose to analyze the antibody depletion during conjugation by running 1-2 µl of the antibody mix before (input) and after (flow-through) the coupling on an SDS-polyacrylamide gel and Coomassie staining.*
    - Finally resuspend the antibody-conjugated magnetic medium in ‘storage solution’ (Recipe B4): add 6.7 μl of storage solution per 1 mg of medium (resulting in a slurry of ~15% w/v) and store at -20 °C. The slurry can be stored in this way for at least 1 year without any noticeable loss of performance.
  2. Affinity purification in conjunction with on-the-beads RNase treatment
A general procedure describing and demonstrating best practices for affinity purification using mammalian cell powder and magnetic media has been previously described (LaCava *et al.*, 2016); a procedure specifically for anti-FLAG affinity capture of ORF2p-3xFLAG-tagged L1 RNPs has also been detailed (Taylor *et al.*, 2016); the following implementation has been modified to test the effects of RNase A/T1 treatment on affinity immobilized L1 RNPs. Before carrying out a SILAC-based quantitative MS experiment (described below), preliminary studies should be carried out using general protein staining and Western blotting (*e.g.*, see Figure 4A, and Taylor *et al.*, 2013, Figure 6).
    - Pre-cool a metal spatula and 8 x 2 ml safe-lock tubes in LN_2_. An aluminum block style tube rack is helpful for this step.
    - Dispense 4 x 200 mg of heavy and light HEK-293T_LD_ cell powders, respectively (*i.e.*, 800 mg total of each), to pre-cooled 2 ml safe-lock tubes.
*
Note: It is important to keep the cell powder frozen. Leave the tubes in LN_2_ until the next step is initiated.
*
    - Place the samples in the rack, open the caps, and let them stand at room temperature for 1 min.
    - Add 800 µl of room temperature protein extraction solution (see Recipes) to each tube.
    - Vortex at maximum speed to fully resuspend the cell powder (should not require more than 30 s), and immediately place the samples on ice.
*Note: Once the cell powder is resuspended, the sample should be held on ice between all manipulations throughout the procedure unless otherwise stated.*
    - Briefly sonicate, on ice or at 4 °C, to completely disperse and homogenize the cell powder. As a guide, we apply ~15-20 J energy per 100 mg cell powder extracted with 400 µl solution (LaCava *et al.*, 2016).
*Note: If clumps are visible, the sonication step should be repeated until no clumps are obvious by visual inspection. Likewise, once extracts appear homogenous upon visual inspection, no further sonication is needed.*
    - Clarify the extract by centrifugation at 20,000 *× g* for 10 min (4 °C).
    - While the samples are in the centrifuge, distribute 20 μl anti-FLAG magnetic medium (slurry) into each of the four 2 ml microcentrifuge tubes and wash thrice each with 1 ml of the extraction solution of choice to thoroughly remove the storage solution.
*Note: We use 5 μl of the beads slurry per 100 mg of the cell powder–this has been titrated for the average expression of ORF2p in our hands, under the preceding conditions. To wash the beads, combine extraction solution with the slurry in a 2 ml microcentrifuge tube and vortex briefly to fully resuspend the beads. Pulse-spin the tube briefly in a mini-centrifuge to collect all the solution at the bottom and then place the tube in a magnetic tube rack until the beads are collected at the side of the tube. Remove the supernatant using a pipette or an aspirator and repeat the washing step twice more time. Perform washing steps at room temperature. After washing, the beads may be held on ice and are ready for use.*
    - Combine all heavy (with heavy) and light (with light) clarified extracts in 5 ml (or larger) tubes, and then dispense each equally into two new 2 ml tubes containing 20 μl anti-FLAG beads resulting in four tubes total: 2 x 400 mg-scale heavy and 2 x 400 mg-scale light ORF2p-3xFLAG affinity isolations.
    - Incubate affinity isolations at 4 °C for 30 min with end-over-end mixing.
    - Wash 3 x 1 ml extraction solution.
    - Transfer the beads to fresh 1.5 ml tubes during 2^nd^ wash.
    - After removing the final wash, organize the tubes for 4 reactions:
      Heavy-untreated: add 50 μl of extraction solution + 2 μl 2 mg/ml BSA
      Heavy-treated: add 50 μl of extraction solution + 2 μl 2 mg/ml RNase A/T1
      Light-untreated: add 50 μl of extraction solution + 2 μl 2 mg/ml BSA
      Light-treated: add 50 μl of extraction solution + 2 μl 2 mg/ml RNase A/T1
    - Incubate the reactions at room temperature for 30 min with gentle mixing (sufficient to keep the beads suspended: ~1,000 rpm).
    - Collect the supernatants after 30 min and save these samples aside. You may choose to analyze them by *e.g.*, analysis by SDS-PAGE (Figure 2A).
    - Wash the beads in each tube with 3 x 1 ml of ice-cold extraction solution.
    - Elute the materials retained on the affinity medium with 40 μl of 1.1x LDS.
*Note: Elution for 10 min at room temperature with moderate agitation is typically sufficient to thoroughly release 3xFLAG-tagged proteins from anti-FLAG M2 epoxy magnetic beads. However, elution may also be achieved by incubation at 70 °C for 5 min with moderate agitation–which is more stringent and effective across a wide variety of affinity media. The latter has been observed to mildly increase the co-elution of immunoglobulin chains from anti-FLAG affinity medium prepared as described in this protocol but has not been observed to significantly contribute to eluted protein content: usually IgG chains are observed at only a bit above the limit of detection by Blue Silver stain (estimated in the ~1-5 ng range). Immunoglobulin chains release from other media may be drastic under these conditions and should be checked, case-by-case.*
    - Samples may be stored at -20 °C or colder.
- Analysis by quantitative MS
  1. Combine elution samples as follows:
    - Light-treated with Heavy-treated (RNase^light^ -with- RNase^heavy^; 10 μl each)
    - Light-untreated with Heavy-untreated (BSA^light^ -with- BSA^heavy^; 10 μl each)
*Note: Samples* ***a*** *and* ***b*** *are controls: the expectation is that light and heavy samples should contribute the comparable protein constituents approximately equally to the mixture–i.e., the metabolic labeling should not itself affect the result. Most proteins observed in these fractions should be ~50% heavy-labeled.*
    - Light-treated with Heavy-untreated (RNase^light^ -with- BSA^heavy^; 30 μl each)
    - Light-untreated with Heavy-treated (BSA^light^ -with- RNase^heavy^; 30 μl each)
*Notes:*
    - *Samples* ***c*** *and* ***d*** *are the experimental samples representing label-swapped technical replicates comparing case (RNase) to control (BSA).*
    - *Small aliquots (~10%) of the separate or combined fractions may be set aside for additional cross-checks by SDS-PAGE and staining or Western blotting.*
The mixing of samples **a-d** is displayed schematically in Figure 4B and the resulting SDS-polyacrylamide gel is shown in Figure 4C.
  2. Add DTT to 25 mM to all samples and heat at 70 °C for 10 min, and then briefly chill samples on ice.
  3. Add iodoacetamide to 100 mM and incubate at room temperature for 30 min in the dark.
  4. Load sample on a 10-well, 1 mm, 4-12% Bis-Tris gel.
    - Before assembling the gel in the electrophoresis apparatus, use a ruler to draw a horizontal line across the gel cassette, in indelible marker, at a position 6 mm below the bottom of the wells.
    - Samples **c** and **d** will be ~70 μl each and may be loaded in two rounds into the same well if needed. First load 40 μl and apply 200 V until the whole sample enters the gel (~30 s-1 min). Now load the remaining sample. Carefully layer the sample at the very bottom of the well using a gel-loading tip; residual glycerol in the well from the first loading will prevent the subsequent sample from “falling” into the well, so layering from the bottom of the well is crucial during the second round of sample loading.
  5. Electrophorese until all the loading dye reaches the 6 mm mark and stain (see Figure 4C), as per Step B3b (above).
  6. Excise gel plugs, and process as per Steps B3c to B3f (above).
  7. LC-MS/MS as per Step B3g (above)
Details of the MS run as we conducted it are as follows: ~3 µl (*i.e.*, 1/3^rd^) were injected per LC-MS/MS analysis. Samples were loaded onto a PicoFrit column (New Objective, Woburn, MA) packed in-house with 6 cm of reverse-phase C18 material (YMC*Gel ODS-A, YMC, Allentown, PA). Peptides were gradient-eluted (Solvent A = 0.1 M acetic acid, Solvent B = 0.1 M acetic acid in 70% v/v acetonitrile, flow rate 200 nl/min) into an LTQ-Orbitrap-Velos (Thermo Fisher Scientific) acquiring data-dependent CID fragmentation spectra.

## Data analysis

**Figure 3.**
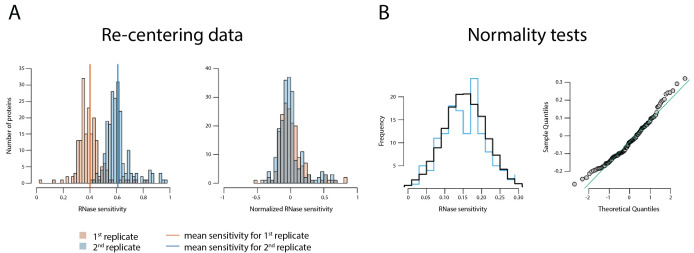
Data treatment. A. Data re-centering as described in step 2a (Data analysis). B. Normality tests as described in step 2b (Data analysis). This figure reproduced from/modified fromTaylor *et al.* (2018).

**Figure 4.**
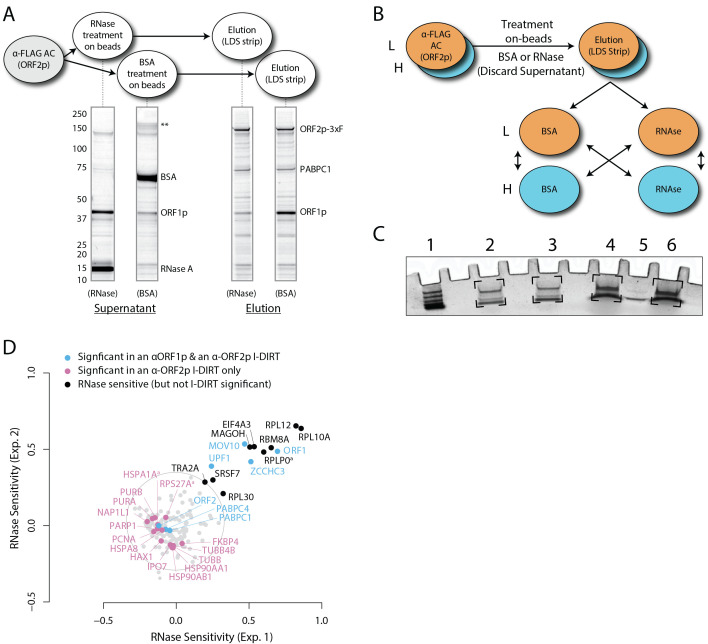
RNase sensitivity affinity capture (modified from Taylor *et al.*, 2018). A. Coomassie Blue G-250 stained SDS-polyacrylamide gel displaying the profiles of the RNase- or BSA-treated affinity immobilized fractions. This kind of gel would be run during preliminary studies of RNase (or other enzyme) treatments. Major changes can be observed visually. If the identities of bands visually observed change upon RNase treatment are not know, they may be cut from *e.g.*, the RNase supernatant lane (far left) and BSA elution lane (far right) and analyzed by mass spectrometry. More, subtle potential differences may be monitored by Western blotting. B. Mixing scheme for light and heavy SILAC-labeled fractions, as described in Step D1. C. Example image of Coomassie Blue G-250 stained gel plugs resulting from Step D5. Brackets indicate the gel areas excised and prepared for analysis. (1) Molecular mass marker. (2) Light-treated with Heavy-treated (RNase^light^ -with- RNase^heavy^). (3) Light-untreated with Heavy-untreated (BSA^light^ -with- BSA^heavy^). (4) Light-treated with Heavy-untreated (RNase^light^ -with- BSA^heavy^). (5) BSA–200 ng. (6) Light-untreated with Heavy-treated (BSA^light^ -with- RNase^heavy^). D. Processed data displaying the degree of RNase-sensitivity (*i.e.*, differential affinity immobilization) exhibited by constituents of the SILAC-labeled fractions. Proteins requiring intact RNA to maintain stable interactions with immobilized ORF2p were released from the RNase-treated medium, while the BSA-treated sample controlled for the spontaneous release of proteins from the medium. Results from the assay have been graphed as the fraction of each detected protein present in the BSA-treated sample (RNase-sensitive proteins are more present in the BSA treated sample), normalized as described in step 2 (Data analysis). A cut-off of *P* = 10^-3^ for RNase-sensitivity is indicated by a light gray circle; proteins that are RNase-sensitive with a statistical significance of *P* < 10^-3^ are outside the circle. Proteins previously ranked significant by I-DIRT analysis (Taylor *et al.*, 2013) are labeled and displayed in blue or magenta (as indicated); black nodes were RNase-sensitive but not significant by I-DIRT; gray, unlabeled nodes were neither RNase-sensitive nor significant by I-DIRT.

1. Initial data processing in MaxQuant
  - Searches were performed against human protein sequences (UP000005640, April 2016) supplemented with custom L1 ORF1p and ORF2p protein sequences, common exogenous contaminants, and a decoy database of reversed protein sequences.
    Search parameters included:
    - Fixed modification: carbamidomethyl (C).
    - Variable modifications: Arg10, Lys8, methionine oxidation.
    - Razor and unique peptides used for protein quantitation.
    - Requantify: enabled.
  - Contaminants, low-scoring proteins, and proteins with only one razor + unique peptide were filtered out from the MaxQuant output file ‘proteingroups.txt’. The list of contaminants was uploaded from the MaxQuant website (http://www.coxdocs.org/; ‘contaminants’). Additionally, proteins with the ‘POTENTIAL CONTAMINANT’ column value ‘+’ were filtered out.
  - Proteins with at least two razor+unique peptides were retained for the analysis. H/(H + L)–*i.e.*, % heavy - and L/(H + L)–*i.e.*, % light - values were derived from unnormalized ‘ratio H/L’ values and were used in post-processing (below) prior to plotting the label-swapped RNase-sensitivity, shown in Figure 4D.
2. Custom post-processing
  - The RNase sensitivity data obtained from MaxQuant were rescaled and normalized such that proteins that did not change upon treatment with RNases were centered at the origin and those that were completely sensitive would give a value of 1.0. In a perfect experiment, unchanging proteins would yield a ratio of 0.5 when comparing the fraction of each protein present in the BSA-treated sample to the sum of both the BSA- and RNase-treated samples; *i.e.*, 1/(1 + 1). However, our data exhibit some variability, as shown in Figure 3A. Therefore, we normalized the data such that the peaks were re-centered at 0.5. From this set, 0.5 was subtracted from the data (centering insensitive proteins at the origin, and completely sensitive proteins at 0.5), followed by multiplication by two to expand the data to cover the range from 0 (insensitive) to 1 (completely sensitive); these latter two transformations are encompassed by the functions:
g(x) = x + b, and f[g(x)] = a (x + b)
where, a = 2; b = -0.5.
  - To determine if these data were normally distributed the distances from the (0, 0) point to protein coordinates were calculated. Proteins with distance less than two median distances were selected. The Shapiro-Wilk normality test (the null-hypothesis of this test is that the population is normally distributed) was applied for the distances (*P*-value = 0.29). The distribution of the distances was plotted as a histogram displaying the frequency (y-axis) versus RNase sensitivity (x-axis) of a simulation of normally distributed data (shown in black) and the actual data shown in blue; a Q-Q plot was also drawn–both displayed in Figure 3B.
  - The mean value and standard deviation were calculated using the distribution of distances from the origin. The distance threshold for *P*-value = 0.001 was calculated using the R programming language. A circle with radius equal to the threshold was plotted and points with distances higher than the threshold were marked as black (Figure 2D).
  - The relevant code for the above post-processing can be obtained here: https://bitbucket.org/altukhov/line-1/src/master/src/protein_analysis/RNAse.R

The prior I-DIRT analysis (Taylor *et al.*, 2013) is an important aspect of the presented analysis. SILAC as conducted here–so-called mix after purification or MAP-SILAC (Wang and Huang, 2008)–does not reveal any information about which interactions are specific or non-specific; it only reveals what has changed between the two treatments (with RNases or BSA). We used I-DIRT, first, in order to establish which interactions are likely to have formed *in vivo*, versus which are likely to be post-extraction *in vitro* artifacts (Tackett *et al.*, 2005). Importantly, the I-DIRT data was collected using comparable experimental conditions (*i.e.*, protein extraction and affinity capture parameters) as for the SILAC data. By using the I-DIRT analysis as a specificity filter on the SILAC analysis, second, we were able to focus our attention on the differential behaviors of proteins most likely to be bona fide physical interactors within L1 RNPs (colored nodes, Figure 4D). Nevertheless, the proteins that were RNase sensitive and not I-DIRT-specific (black nodes, Figure 4D) proved informative: they are all RNA-binding proteins that one might expect to be released by RNase treatment. Thus, they served as a secondary indicator that the treatment is effective. Notably, PABPC1 and C4–poly(A) RNA binding proteins appear insensitive to RNase treatment. This is most likely due to the fact that neither RNase A nor T1 cleave RNA at adenosine residues (Volkin and Cohn, 1953; Yoshida, 2001); hence poly(A) binding proteins may not be ready targets for release from direct RNA binding by the assay implemented here (or generally, using these ribonucleases) – see further discussion in (Taylor *et al.*, 2018). On the basis of these data, we concluded that UPF1, ZCCHC3, MOV10, and ORF1p are at least partly dependent upon the presence of intact RNA to remain stably associated within L1 RNPs.

## Recipes

- Cell culture media
*
Note: Phenol red (final 0.001% w/v) may be added as a pH indicator. Antibiotics and/or antimycotics are not recommended in combination with PEI-based transfection; the combination of PEI and penicillin-streptomycin reduces cell viability and transfection efficiency. Most of our cultures are antibiotic free. Although the Freestyle-293 medium is designed to be used serum-free with the manufacturer’s cell lines, we have seen reduced growth rates and low viability at FBS below 1% (v/v) for HEK-293T_LD_ and other 293 cells.
*
  1. Heavy SILAC medium
    Supplement Freestyle-293 medium lacking arginine and lysine with:
    1% v/v dialyzed FBS
    2 mM L-glutamine
    200 mg/L L-proline
800 mM ^13^C_6_^15^N_4_-L-arginine (Arg10)
400 mM ^13^C_6_^15^N_2_-L-lysine (Lys8)
  2. Light SILAC medium
    Supplement Freestyle-293 medium lacking arginine and lysine with:
    1% v/v dialyzed FBS
    2 mM L-glutamine
    200 mg/L L-proline
    800 mM L-arginine
    400 mM L-lysine
- Magnetic bead coupling solutions
  1. 10x PBS stock solution
3.1 g of NaH_2_PO_4_·H_2_O, 10.9 g of Na_2_HPO_4_, and 87.8 g NaCl per liter; the final pH should be 7.4, confirm with pH indicator paper
    A 1x solution yields ~10 mM phosphate, 150 mM NaCl at pH 7.4
  2. 0.1 M sodium phosphate pH 7.4
Follow a standard phosphate buffer chart, or *e.g.*, add 3.1 g of NaH_2_PO_4_·H_2_O and 10.9 g of Na_2_HPO_4_ (anhydrous) to distilled H_2_O to make a volume of 1 L
    The pH of the final solution will be 7.4–confirm with pH indicator paper
  3. 3 M Ammonium Sulfate
39.6 g (NH_4_)_2_SO_4_ combined with 0.1 M sodium phosphate pH 7.4 to a final volume of 100 ml
  4. Magnetic Media Storage Solution
    1x PBS [final] containing 0.5 mg/ml BSA and 50% (v/v) glycerol
- Protein extraction, staining, and destaining solutions
  1. Protein extraction solution
    20 mM HEPES-Na pH 7.4
    1% (v/v) Triton X-100
    500 mM NaCl
*Note: This solution is supplemented with protease inhibitors during protein extraction and is used without the addition of protease inhibitors during subsequent washes of the affinity medium. The frozen cell powders should be extracted with a room temperature solution, resulting in cold extracts that are subsequently handled on ice and/or at 4 °C; for subsequent washes of the affinity medium, after protein capture, the solution should be ice-cold.*
  2. Gel acidification solution
    0.1 M HCl
  3. Blue Silver Stain (modified from Candiano *et al.*, 2004)
20% (w/v) H_3_PO_4_
10% (w/v) (NH_4_)_2_SO_4_
    0.12% (w/v) Coomassie Brilliant Blue G-250
    20% (v/v) methanol
    - To water (1/10^th^ of the final volume), add first the phosphoric acid (we use double the published quantity). Note, commercially available concentrated phosphoric acid tends to be prepared at ~85% (w/w) with a density of ~1.69 g/ml.
    - Next, add the ammonium sulfate. When the ammonium sulfate has dissolved (incrementally add more water if this is taking excessively long), add the G-250 dye, and bring the water to 80% of the final volume.
    - To this solution, under vigorous stirring, add anhydrous methanol to 20% the final volume; once the solution is thoroughly mixed, adjust it to the final volume with water.
    - This solution should be kept in a brown or foil-covered bottle and is stable at room temperature for 6 months.
  4. Gel plug destaining solution
    50 mM ammonium bicarbonate in 50% (v/v) acetonitrile, prepared in LC-MS grade water
*Example: Combine one part 100 mM ammonium bicarbonate* *in LC-MS grade water with one part LC-MS grade acetonitrile. Prepare this solution fresh before use. One-hundred millimolar ammonium bicarbonate yields a solution of pH* ≥ *8.5; 50 mM ammonium bicarbonate should produce a solution of pH ~8.*
- OMIX C18 tip, peptide desalting solution
  1. Tip wetting solution
    0.1% (w/v) TFA
    50% (v/v) acetonitrile
    200 µl per sample
  2. Tip wash solution
    0.1% (w/v) TFA
    600 µl per sample
  3. Elution 1 solution
    0.5% (v/v) acetic acid
    40% (v/v) acetonitrile
    80 µl per sample
  4. Elution 2 solution
    0.5% (v/v) acetic acid
    80% (v/v) acetonitrile
    80 µl per sample
